# In Vitro and Embryonic Evaluation of Recombinant *Larus ridibundus* IFN-α and Mx Proteins Against Newcastle Disease Virus

**DOI:** 10.3390/pathogens15080818

**Published:** 2026-08-03

**Authors:** Hua Chang, Shaoxia Pu, Mingxiu Yuan, Yi Chen, Shengjie Ren, Hongli Zhang, Gang Duan, Feiyan Dai, Xun Xiang

**Affiliations:** College of Veterinary Medicine, Yunnan Agricultural University, Kunming 650201, China; changhuaxx@126.com (H.C.);

**Keywords:** *Larus ridibundus*, Newcastle disease virus, IFN-α, Mx, qRT-PCR, innate antiviral immunity

## Abstract

Newcastle disease virus (NDV) is an important avian pathogen that can circulate in migratory birds, including *Larus ridibundus*. This study examined the transcriptional responses and anti-NDV activities associated with recombinant IFN-α and Mx proteins from *L. ridibundus*. The IFN-α and Mx open reading frames were cloned (GenBank: OP263971 and OP263970), expressed using a pET32a(+)/*E. coli* system, and assessed using chicken embryos, DF-1 cells, and primary gull lymphocytes. qRT-PCR analysis showed that NDV infection increased IFN-α and Mx transcript abundance in gull lymphocytes, with peak levels at 48 h post-infection (*p* < 0.01). In chicken embryos, recombinant IFN-α was associated with higher embryo survival and lower NDV NP transcript abundance than recombinant Mx under the tested conditions. In DF-1 cells and primary gull lymphocytes, 0.25 mg/mL recombinant IFN-α was associated with reduced NDV NP transcript abundance, and morphological inspection suggested less severe cytopathic effects in DF-1 cells. IFN-α treatment was also accompanied by lower transcription of TLR7, MyD88, IRF7, and Mx compared with the NDV group at 48 h. These data support a role for gull IFN-α in limiting NDV-associated transcriptional responses in vitro, while further protein-level and infectious-virus assays are required to define the underlying mechanism.

## 1. Introduction

Newcastle disease virus (NDV) is an important avian paramyxovirus with a single-stranded negative-sense RNA genome. Virulent NDV strains can cause severe respiratory, digestive, and neurological disease in poultry and can lead to high mortality in susceptible flocks [1]. Although rare human conjunctivitis has been reported after occupational exposure, NDV is primarily an avian pathogen and its public health significance should be interpreted cautiously [2].

*Larus ridibundus* is a globally distributed migratory bird with migration routes that overlap with wetlands, poultry farms, and human settlements. NDV carried in feces or secretions from migratory birds may contribute to environmental contamination and cross-regional dissemination [3,4]. Migratory birds can also contribute to viral maintenance and evolution through movement, host switching, mutation, and selection. Therefore, characterizing innate antiviral responses in *L. ridibundus* may help clarify host–virus interactions in wild birds and inform NDV surveillance.

Innate antiviral immunity is initiated by pattern-recognition receptors, including Toll-like receptors (TLRs), which detect pathogen-associated molecular patterns and activate downstream immune responses [5,6,7,8]. During RNA virus infection, endosomal TLR7 can recognize viral single-stranded RNA and signal through MyD88 to activate interferon regulatory factors, including IRF7, leading to type I interferon induction [9,10,11,12,13,14]. Recent studies indicate that IFN induction is regulated by additional host factors and is not a simple linear cascade [15]. After secretion, IFN-α exerts antiviral effects mainly through IFNAR-dependent JAK1/TYK2 and STAT1/STAT2 signaling, ISGF3 formation, and the induction of interferon-stimulated genes (ISGs), including Mx [16,17,18,19]. Mx proteins are interferon-induced antiviral GTPases whose activity can be host-, virus-, and protein-context dependent [17,20,21,22]. However, the transcriptional behavior of IFN-α and Mx in NDV-infected *L. ridibundus* lymphocytes remains poorly characterized.

The TLR7 (MZ668652), MyD88 (OR145974), and IRF7 (OR145972) genes of *L. ridibundus* were obtained in previous work. In the present study, we cloned the IFN-α and Mx genes, prepared recombinant proteins in vitro, and evaluated their anti-NDV activity in chicken embryos, DF-1 cells, and primary *L. ridibundus* lymphocytes. We focused on NDV NP transcript abundance, host immune-gene transcription, and qualitative cytopathic-effect observations as readouts. The study was designed to characterize transcriptional associations and preliminary antiviral activity rather than to prove complete pathway activation or functional dependence.

## 2. Materials and Methods

In November 2025, peripheral blood samples (5 mL per bird) were collected from 30 healthy adult *Larus ridibundus* around Dianchi Lake, Kunming, Yunnan Province, via sterile heparin anticoagulant tubes. Nine-day-old chicken embryos were purchased from Beijing Weitong Lihua Experimental Animal Technology Co., Ltd., Beijing, China. The embryos were incubated in a fully automated incubator at 37 °C with 60% humidity.

Expression and purification of recombinant proteins: The peripheral blood lymphocytes of *Larus ridibundus* were isolated via density gradient centrifugation. Total RNA was extracted via TRIzol reagent. RNA purity was assessed via A260/A280 ratios ranging between 1.8 and 2.0. The primers were designed to amplify full-length IFN-α and Mx ORFs (Table 1), which were inserted into pMD18-T, sequenced and submitted to GenBank.

After double digestion of the recombinant T-vector, target fragments were linked with pET32a(+) to construct prokaryotic expression plasmids. The recombinant expression plasmids were transformed into Transetta (DE3) competent cells. After cultivation to an OD600 of 0.6–0.8, IPTG induction parameters were optimized by SDS-PAGE. IPTG at different concentrations, induction temperatures, and induction durations was tested. After ultrasonication, soluble and insoluble fractions were examined by SDS-PAGE, and the target proteins were purified using Ni^2+^ affinity chromatography after the selected expression and refolding procedure. Protein concentration was determined by the BCA method, and protein size and purity were evaluated by SDS-PAGE.

Determination of IFN-α and Mx gene transcription levels in lymphocytes incubated with NDV: *L. ridibundus* lymphocytes were isolated and adjusted to 5 × 10^6^ cells/mL. The NDV stock was titrated by the Reed–Muench method and the TCID50 was calculated. Cells were collected at 12, 24, 48, 60, and 72 h after NDV exposure to detect IFN-α and Mx transcription. Primers for IFN-α, Mx, and the internal reference gene β-actin are listed in Table 1. Gene transcription was measured by qRT-PCR using the reaction system and cycling program shown in Table 2 and Table 3.

Analysis of the anti-NDV activity of recombinant IFN-α and Mx proteins in chicken embryos: The antiviral proteins were diluted with sterile normal saline to 0.25 mg/mL. Chicken embryo experiments were performed using three treatment modes: pre-treatment (protein administration before viral inoculation), co-incubation control (protein and virus mixed before inoculation), and post-infection treatment (protein administration after viral inoculation). A virus control group inoculated only with NDV and a blank control group supplemented only with normal saline or diluent were also established. The experiment included 20 chicken embryos per group and three replicate measurements for each treatment mode. Embryos were incubated at 37 °C for 48 h, after which brain tissue samples were collected for qRT-PCR analysis of relative NDV NP transcript abundance.

Analysis of the anti-NDV activity of recombinant IFN-α protein in DF-1 cells: IFN-α protein was diluted to 0.25 mg/mL and tested using pre-treatment, co-incubation control, and post-infection treatment modes. The three assays were defined as protein addition before virus inoculation, virus–protein co-incubation before inoculation, and protein addition after virus inoculation, respectively. Cells were collected at 8, 24, 48, and 72 h, and relative NDV NP transcript abundance was detected by qRT-PCR. For dose comparison under post-infection treatment conditions, high (0.5 mg/mL), medium (0.25 mg/mL), and low (0.1 mg/mL) IFN-α doses were tested.

Analysis of the anti-NDV activity of recombinant IFN-α protein in lymphocytes: *L. ridibundus* lymphocytes were isolated, and the selected 0.25 mg/mL post-infection IFN-α treatment condition was used for lymphocyte assays. Cells were harvested at 8, 24, 48, and 72 h post-treatment to detect changes in NDV NP transcription and in host TLR7, MyD88, IRF7, and Mx transcription. The 48 h time point was used for the principal between-group comparisons because the time-course experiments showed peak or near-peak NDV NP and host-gene transcript abundance at this point. Relative transcript abundance was calculated using the 2^−ΔΔCt^ method. GraphPad Prism 9.5 was used for data visualization and statistical analysis. Each quantitative condition contained three replicate measurements (*n* = 3); individual values are shown in the figures, and summary data are presented as mean ± SD. The replicate type could not be classified consistently as biological or technical across all assays; therefore, the neutral term replicate measurements is used rather than assigning an unsupported classification. Single-factor comparisons among three or more groups or time points were analyzed by ordinary one-way ANOVA followed by Tukey’s multiple-comparison test. Treatment-by-time datasets were analyzed by ordinary two-way ANOVA followed by Tukey’s test. For the prespecified virus-versus-IFN-α comparisons at 48 h in the lymphocyte assays, two-sided Welch’s *t*-tests were used, with Holm adjustment across the five displayed endpoints. Parametric analyses assume independent observations, approximately normal residuals, and variance homogeneity where applicable. Because n = 3 provides limited power for formal assumption testing, individual values are displayed and inferential results are interpreted cautiously. *p* < 0.05 was considered significant, and *p* < 0.01 was considered highly significant.

## 3. Results

### 3.1. Expression and Purification of L. ridibundus IFN-α and Mx Recombinant Proteins

**Figure 1:**
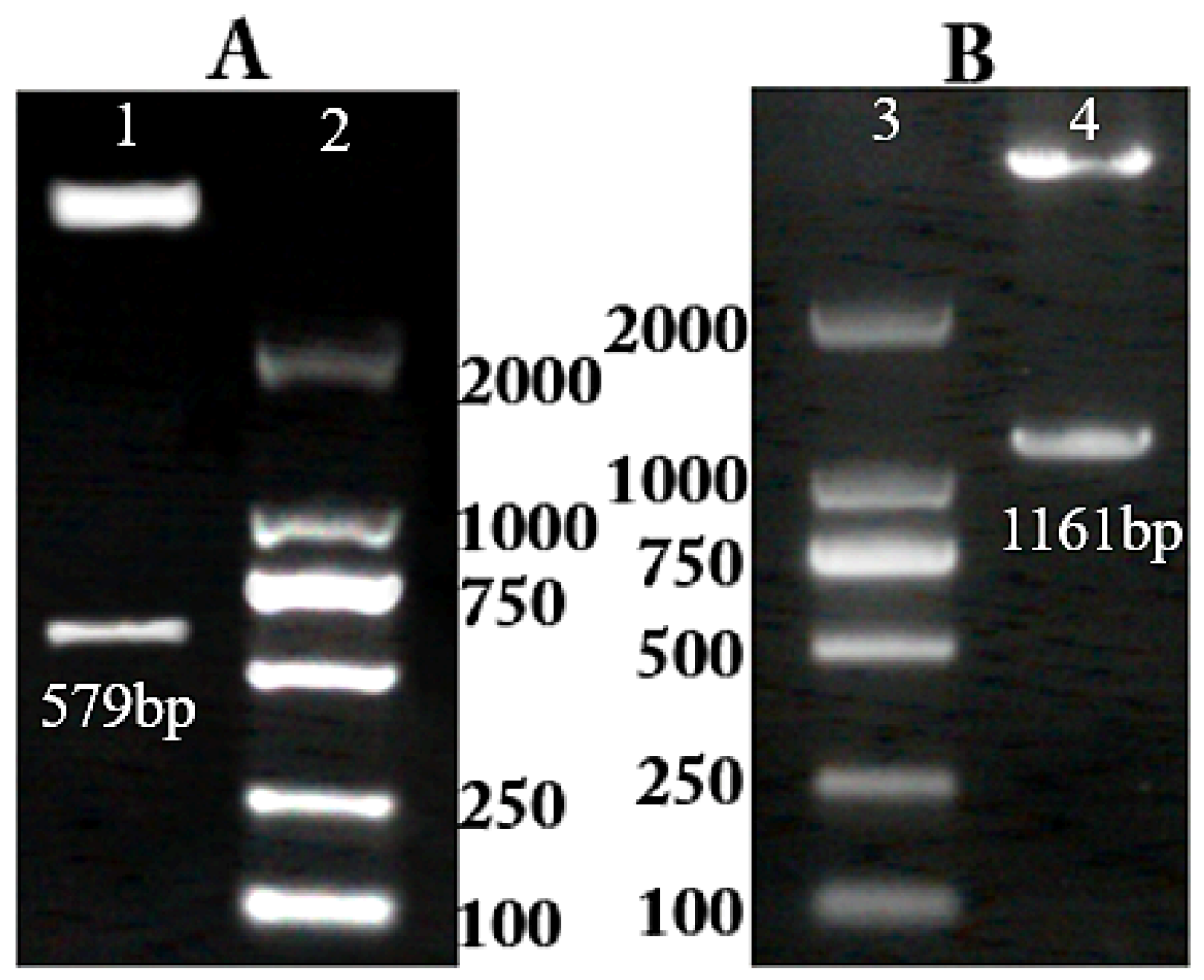
Identification of Recombinant IFN-α and Mx Plasmids by Restriction Enzyme Digestion. (**A**): Identification of Recombinant IFN-α1; 1: pET-32a-IFN-α plasmid; 2: DL2000 marker. (**B**). Identification of Recombinant Mx; 3. DL2000 marker; 4. pET-32a-Mx plasmid.

Following 48 h of stimulation of *Larus ridibundus* lymphocytes with concanavalin A, total RNA was extracted via the TRIzol method. PCR amplification yielded gene fragments of IFN-α and Mx, the sizes of which were consistent with theoretical expectations. After purification, the fragments were cloned and inserted into the pMD18-T vector, and the sequence correctness was verified by sequencing. These sequences were submitted to GenBank with the following accession numbers: IFN-α (OP263971) and Mx (OP263970). T-clone recombinant plasmids (pMD18-T-IFN-α and pMD18-T-Mx) were constructed and confirmed by restriction enzyme digestion. The target fragments and pET-32a (+) vector were subsequently double-digested with EcoRI and XhoI, ligated, and transformed to obtain positive prokaryotic expression vectors, which were designated pET-32a-IFN-α and pET-32a-Mx. The product sizes were consistent with expectations, the fragment sizes of IFN-α (Figure 1 lane 1) and Mx (Figure 1 lane 4) were 579bp and 1161 bp, respectively. In addition, the plasmid was sequenced and verified by Takara Biomedical Technology (Dalian, China), and the sequence was confirmed to be correct, supporting subsequent protein expression experiments.

**Figure 2:**
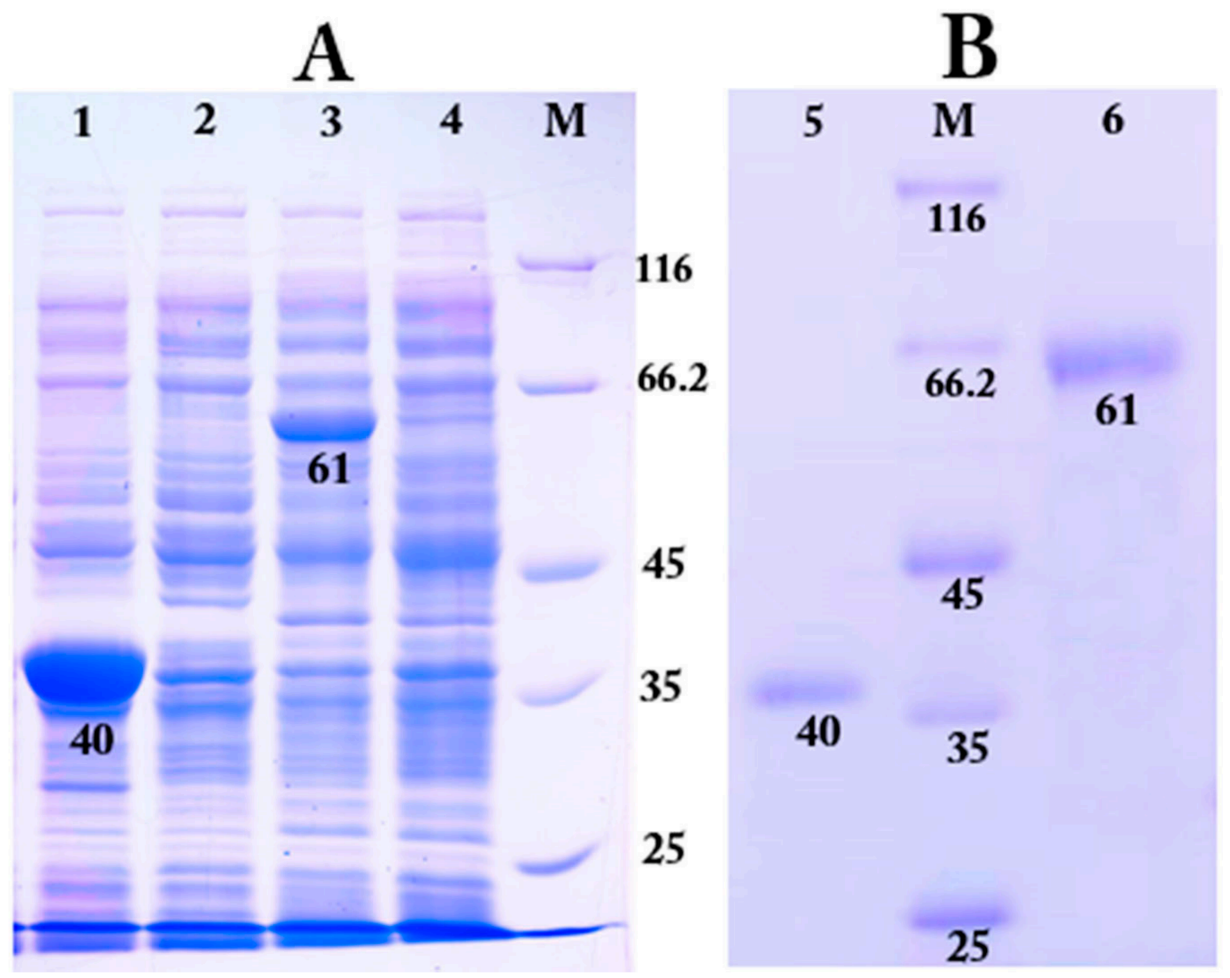
Results of the expression and purification of the recombinant IFN-α and Mx protein. (**A**). The expression of the recombinant IFN-α and Mx proteins. 1: Induced group of the recombinant IFN-α. 2: Non-induced group of the recombinant IFN-α. 3: Induced group of the recombinant Mx; 4: Non-induced group of the recombinant Mx; M, protein marker. (**B**). The purification of the recombinant IFN-α and Mx proteins. 5: IFN-α protein purification; M, protein marker; 6: Mx protein purification.

All recombinant expression plasmids were transformed into Transetta (DE3) competent cells. After IPTG induction, SDS-PAGE analysis showed no obvious target band in the non-induced group, whereas the induced group showed bands consistent with the expected molecular weights. The optimized induction condition was 1 mmol/L IPTG at 37 °C for 5 h. After large-scale cultivation and ultrasonication, the target proteins were mainly detected in the precipitate fraction. Following purification by Ni column chromatography and gradient renaturation, BCA-determined protein concentrations were 1.3 mg/mL for IFN-α (approximately 40 kDa; Figure 2A, lane1) and 1.247 mg/mL for Mx (approximately 61 kDa; Figure 2A, lane3). SDS-PAGE supported the expected sizes of the purified proteins (Figure 2B).

### 3.2. Transcriptional Changes in IFN-α and Mx Genes in L. ridibundus Lymphocytes Infected with NDV

**Figure 3:**
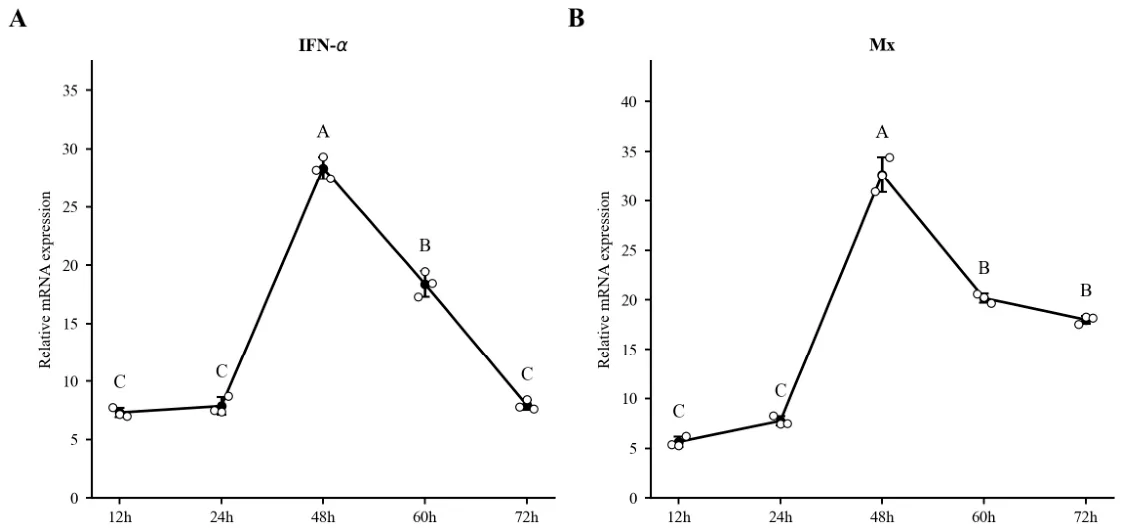
Changes in IFN-α and Mx transcription in *L. ridibundus* lymphocytes infected with NDV. (**A**): IFN-α transcript abundance; (**B**): Mx transcript abundance. Data are shown as mean ± SD with individual replicate values overlaid (n = 3 replicate measurements per time point). Time points sharing the same uppercase letter are not significantly different (*p* ≥ 0.05), whereas different uppercase letters indicate *p* < 0.05 (ordinary one-way ANOVA followed by Tukey’s test).

The TCID_50_ of NDV determined by the Reed–Muench method was 10^−3.762^/0.2 mL. After *L. ridibundus* lymphocytes were exposed to NDV, IFN-α and Mx transcript abundance increased over time and peaked at 48 h post-infection. IFN-α transcription increased first and then decreased from 48 to 72 h (Figure 3A). Mx transcription showed a similar increase to 48 h and remained relatively high at 72 h; the difference between 60 and 72 h was not significant (*p* > 0.05) (Figure 3B). These results indicate that NDV infection was associated with time-dependent induction of IFN-α and Mx transcripts in gull lymphocytes.

### 3.3. Anti-NDV Activity of Recombinant IFN-α and Mx Proteins in Chicken Embryos

**Figure 4:**
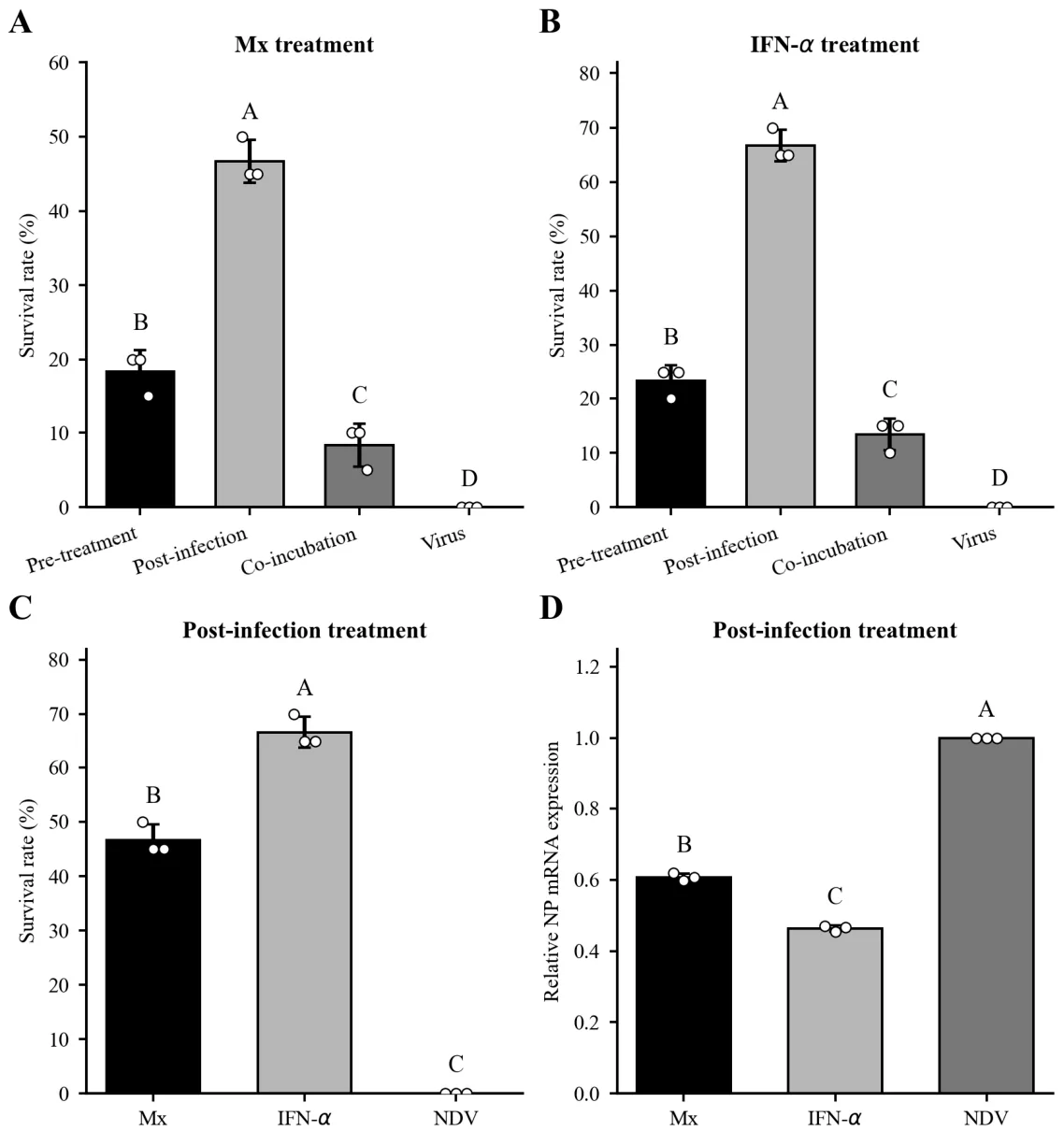
Anti-NDV activity of recombinant IFN-α and Mx proteins in chicken embryos. (**A**): Survival rate after Mx treatment; (**B**): survival rate after IFN-α treatment; (**C**): survival rate in the post-infection treatment group; (**D**): NDV NP transcript abundance under post-infection treatment conditions. Pre-treatment denotes protein administration before virus inoculation, post-infection treatment denotes protein administration after virus inoculation, and co-incubation control denotes protein-virus mixing before inoculation. Data are shown as mean ± SD with individual replicate values overlaid (n = 3 replicate measurements per condition). Within each panel, groups sharing the same uppercase letter are not significantly different (*p* ≥ 0.05), whereas different uppercase letters indicate *p* < 0.05 (ordinary one-way ANOVA followed by Tukey’s test).

To evaluate the anti-NDV activities of recombinant IFN-α and Mx proteins (0.25 mg/mL) in chicken embryos, embryo survival and NDV NP transcript abundance were compared across treatment modes. Both proteins showed the most favorable results in the post-infection treatment group compared with the pre-treatment and co-incubation control groups (Figure 4A,B). Under post-infection treatment conditions, embryo survival was higher in the IFN-α group than in the Mx group, and the difference was highly significant (*p* < 0.01) (Figure 4C). NDV NP transcript abundance in the IFN-α and Mx treatment groups was lower than in the NDV group (*p* < 0.01), and the IFN-α group showed lower NP transcript abundance than the Mx group (*p* < 0.01) (Figure 4D). These data support a stronger antiviral association for recombinant IFN-α than recombinant Mx in the measured chicken embryo endpoints.

### 3.4. Anti-NDV Activity of Recombinant IFN-α Protein in DF-1 Cells

**Figure 5:**
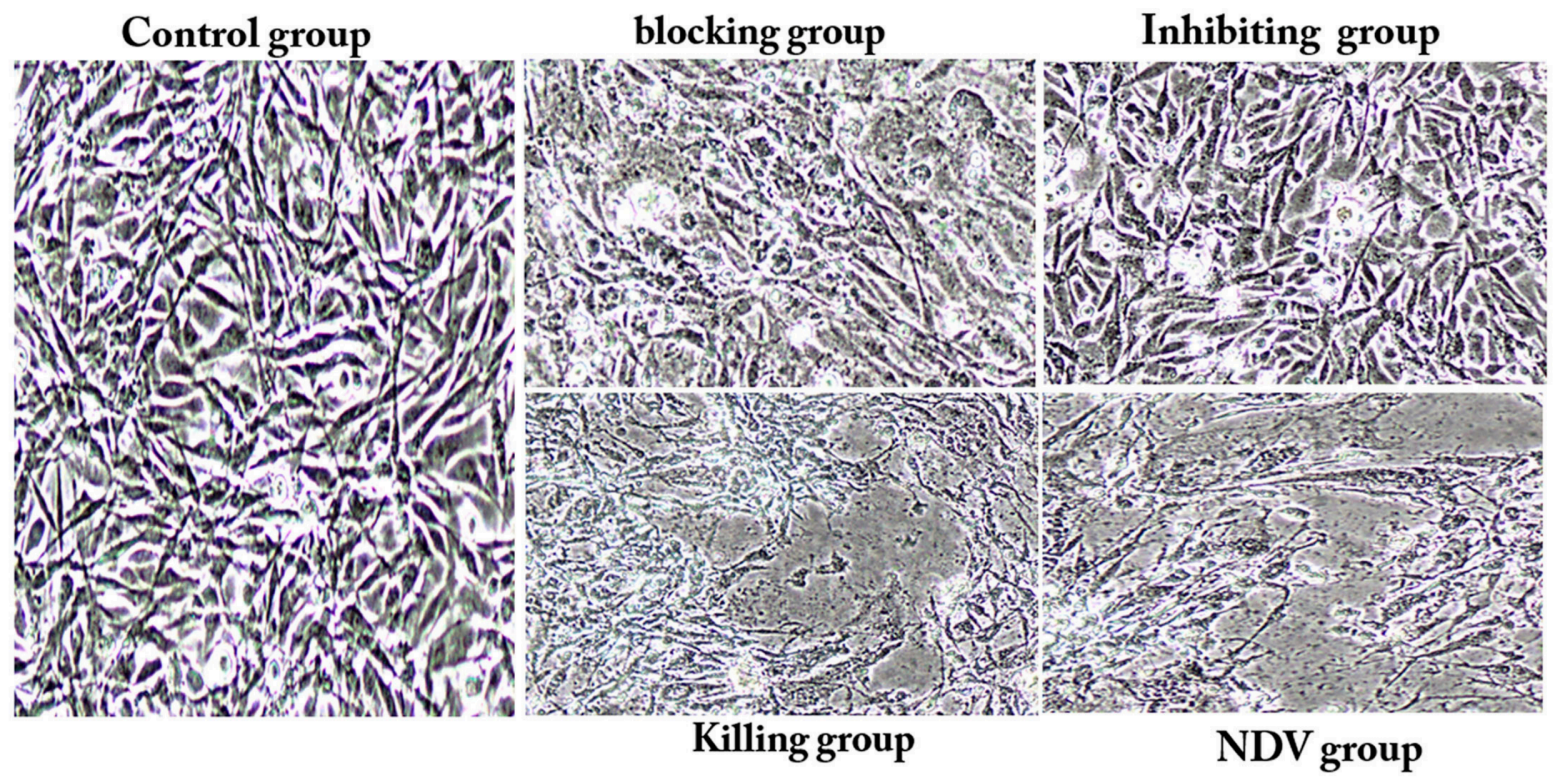
Cytopathic effects in DF-1 cells treated with recombinant IFN-α protein under different treatment modes. In the microscopy composite, blocking, inhibiting, and killing denote pre-treatment, post-infection treatment, and co-incubation control, respectively. Compared with the NDV group, morphological inspection suggested less severe cytopathic effects after IFN-α treatment, with the clearest qualitative difference observed in the post-infection treatment group (magnification: 20 × 10).

**Figure 6:**
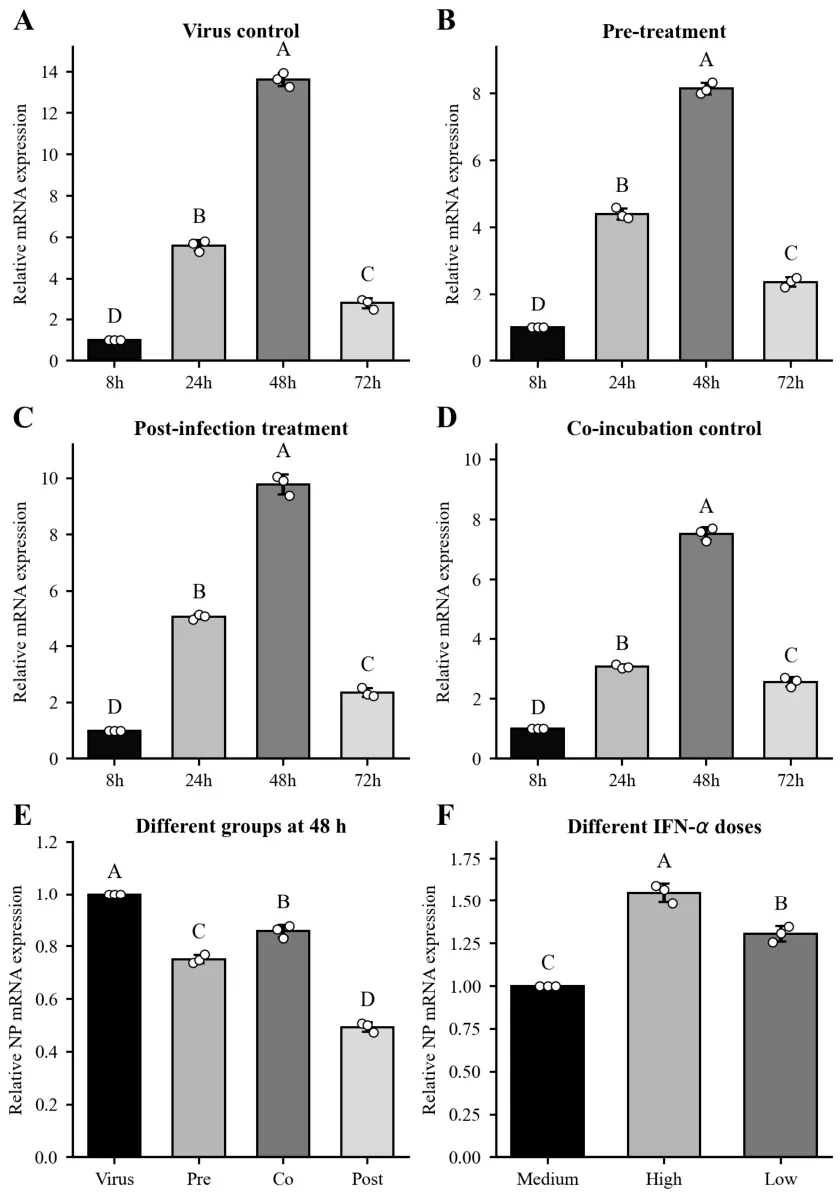
Anti-NDV activity of IFN-α under different treatment modes and doses in DF-1 cells. (**A**): NDV NP transcript abundance in the virus control group; (**B**): NP transcript abundance in the pre-treatment group; (**C**): NP transcript abundance in the post-infection treatment group; (**D**): NP transcript abundance in the co-incubation control group; (**E**): NP transcript abundance in different groups at 48 h; (**F**): NP transcript abundance after different IFN-α doses. In panel E, Pre, Co, and Post denote pre-treatment, co-incubation control, and post-infection treatment, respectively. Data are shown as mean ± SD with individual replicate values overlaid (n = 3 replicate measurements per condition). Within each panel, groups sharing the same uppercase letter are not significantly different (*p* ≥ 0.05), whereas different uppercase letters indicate *p* < 0.05 (ordinary one-way ANOVA followed by Tukey’s test).

Based on the chicken embryo results, recombinant IFN-α was selected for further antiviral analysis in DF-1 cells. IFN-α protein (0.25 mg/mL) was evaluated using pre-treatment, co-incubation control, and post-infection treatment modes. Antiviral activity was assessed by qRT-PCR analysis of NDV NP transcript abundance and by observation of cytopathic effects (CPE) at 48 h. Compared with the NDV group, morphological inspection suggested less severe CPE in IFN-α-treated cells, with the clearest qualitative difference in the post-infection treatment group (Figure 5). NDV NP transcript abundance increased first and then decreased from 8 to 72 h and peaked at 48 h in all groups (Figure 6A–D). At 48 h, NP transcript abundance differed significantly among the four groups, and the post-infection treatment group showed lower NP transcript abundance than the virus control, pre-treatment, and co-incubation control groups (*p* < 0.01) (Figure 6E). Dose comparison under post-infection treatment conditions showed the lowest NP transcript abundance in the 0.25 mg/mL IFN-α group (*p* < 0.01) (Figure 6F).

### 3.5. IFN-α-Associated Changes in NDV NP and Host Immune-Gene Transcription in L. ridibundus Lymphocytes

IFN-α-associated transcriptional changes were assessed in *L. ridibundus* lymphocytes using 0.25 mg/mL IFN-α under post-infection treatment conditions. NDV NP and host TLR7, MyD88, IRF7, and Mx transcript abundance were detected by qRT-PCR.

**Figure 7:**
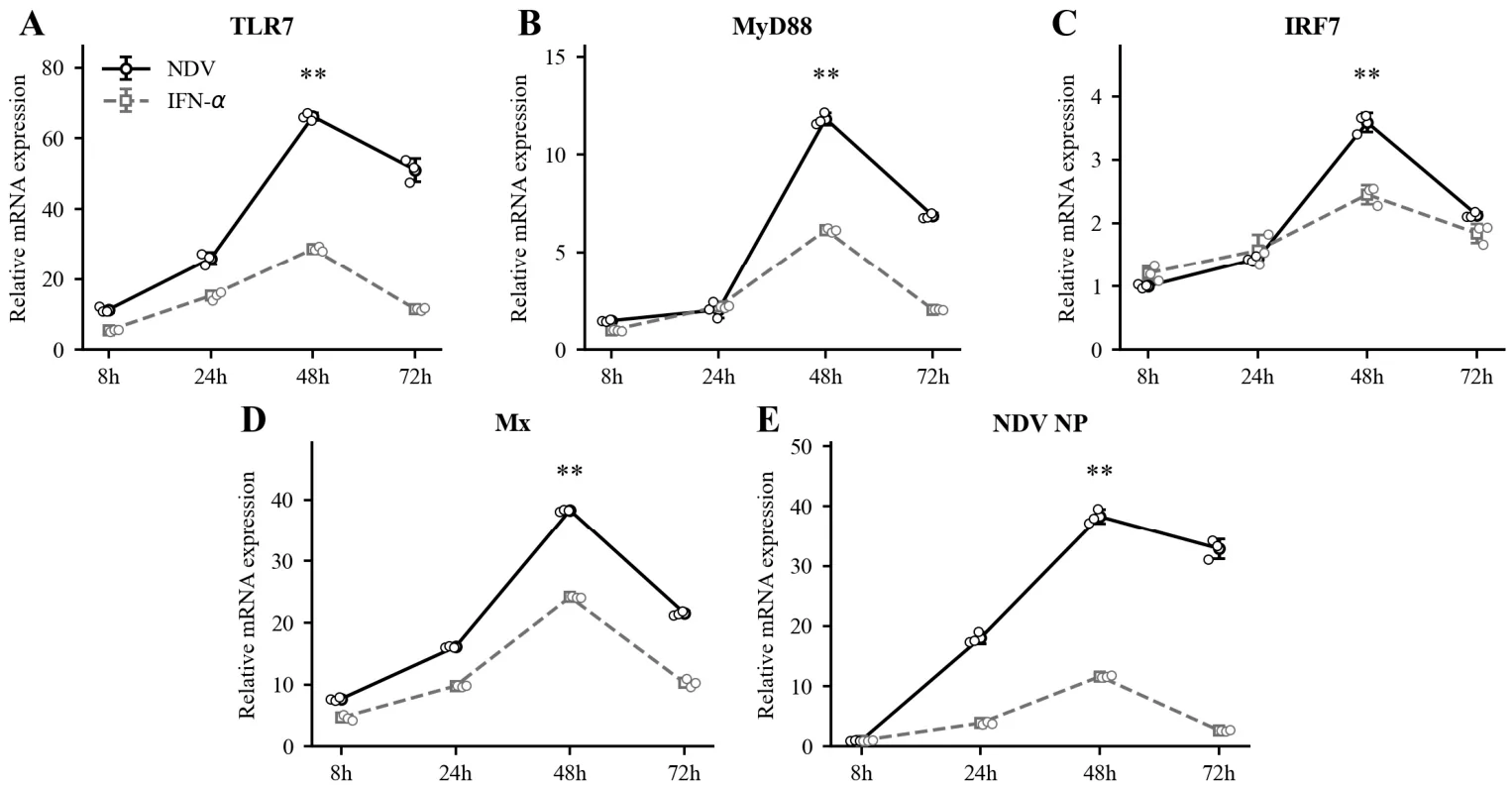
Gene transcription changes in *L. ridibundus* lymphocytes after recombinant IFN-α treatment. (**A**): TLR7 transcript abundance; (**B**): MyD88 transcript abundance; (**C**): IRF7 transcript abundance; (**D**): Mx transcript abundance; (**E**): NDV NP transcript abundance. Data are shown as mean ± SD with individual replicate values overlaid (*n* = 3 replicate measurements per condition). At 48 h, the virus and IFN-α-treated groups were compared using two-sided Welch’s *t*-tests with Holm adjustment across the five displayed endpoints; two asterisks indicate *p* < 0.01.

NDV NP transcript abundance increased first and then decreased, with a peak at 48 h. At 48 h, NP transcript abundance in the IFN-α-treated group was significantly lower than in the virus group after Holm adjustment (*p* < 0.01) (Figure 7E). TLR7 (Figure 7A), MyD88 (Figure 7B), IRF7 (Figure 7C), and Mx (Figure 7D) transcription also increased first and then decreased over time. At 48 h post-infection, the transcript abundance of TLR7, MyD88, IRF7, and Mx was lower in the IFN-α-treated group than in the virus group after Holm adjustment (*p* < 0.01). These results indicate that IFN-α treatment was associated with lower NDV NP transcript abundance and reduced transcription of selected host immune genes at the 48 h time point.

## 4. Discussion

*L. ridibundus* is a migratory wild bird species that may contribute to the movement and maintenance of avian viruses, including NDV. Direct in vivo infection studies in this species are constrained by ethical, conservation, and practical considerations. Therefore, this study used chicken embryos, DF-1 cells, and primary *L. ridibundus* lymphocytes to evaluate recombinant IFN-α and Mx proteins. These models provide preliminary information on anti-NDV activity but do not fully reproduce the biology of the natural host.

The three experimental systems served different purposes. The chicken embryo model was used for preliminary comparison of recombinant IFN-α and Mx based on embryo survival and NDV NP transcript abundance. DF-1 cells were used to further characterize recombinant IFN-α treatment mode and dose. Primary *L. ridibundus* lymphocytes were used to examine host-derived transcriptional responses after IFN-α treatment. Because chicken embryos and DF-1 cells are heterologous surrogate systems, the results should be interpreted as screening-level evidence and not as complete proof of species-specific antiviral mechanisms in gulls.

The recombinant IFN-α and Mx proteins were produced using an *E. coli* expression system, which is efficient and suitable for producing recombinant proteins for preliminary functional assays. BCA quantification and SDS-PAGE confirmed the expected protein concentrations and approximate molecular weights of IFN-α (1.3 mg/mL; approximately 40 kDa) and Mx (1.247 mg/mL; approximately 61 kDa). However, SDS-PAGE alone does not fully demonstrate biological activity, folding state, or the absence of bacterial contaminants. Because the proteins were produced in *E. coli*, endotoxin removal and quantification are important for future validation, especially for experiments measuring innate immune transcripts.

NDV infection increased IFN-α and Mx transcript abundance in *L. ridibundus* lymphocytes, with both genes reaching peak transcript levels at 48 h post-infection. This pattern is consistent with activation of innate antiviral transcriptional responses after NDV exposure. However, the present qRT-PCR data do not demonstrate IFN-α secretion, IRF7 phosphorylation, MyD88 recruitment, or functional dependence on the TLR7/MyD88/IRF7 pathway. Accordingly, the results should be interpreted as transcriptional associations rather than direct evidence that the complete pathway is activated or required.

In chicken embryos, recombinant IFN-α and Mx were compared using embryo survival and NDV NP transcript abundance. The post-infection treatment mode produced the most favorable results among the tested modes. Within these endpoints, recombinant IFN-α showed a stronger association with embryo survival and lower NDV NP transcript abundance than recombinant Mx. This comparison should be limited to the chicken embryo readouts because equivalent downstream mechanistic analyses for recombinant Mx were not performed in DF-1 cells or primary gull lymphocytes. The biological interpretation of extracellular recombinant Mx also requires caution, because Mx is classically an intracellular interferon-stimulated antiviral GTPase.

In DF-1 cells, the terminology of the three treatment modes was revised to avoid implying direct virucidal activity of IFN-α. The co-incubation control tests whether protein-virus mixing before inoculation affects subsequent readouts, but interferons are not expected to directly inactivate virions. The lowest NDV NP transcript abundance was observed after post-infection IFN-α treatment, suggesting that IFN-α-mediated cellular responses after infection may contribute to the observed reduction in NP transcripts. Because infectious virus titration, plaque assay, immunofluorescence, and viral-protein immunoblotting were not performed, reduced NP transcript abundance should not be overinterpreted as a complete demonstration of reduced infectious-virus production.

The IFN-α dose comparison indicated that 0.25 mg/mL was associated with the lowest NDV NP transcript abundance under the tested post-infection treatment conditions. This non-linear dose response may reflect the experimental system and should be confirmed with additional functional assays. CPE observation provided supportive morphological information, but the study did not include a blinded lesion-scoring system or non-parametric analysis of ordinal CPE scores. Therefore, the CPE results should be interpreted as qualitative observations and cannot substitute for direct measurement of infectious viral particles or quantitative pathological assessment.

Primary *L. ridibundus* lymphocytes were used to reduce, but not eliminate, the limitations of heterologous surrogate systems. In this host-derived cell model, IFN-α treatment was associated with lower NDV NP transcript abundance compared with the virus group. TLR7, MyD88, IRF7, and Mx transcripts also decreased in the IFN-α-treated group at 48 h. One possible explanation is that lower viral RNA levels after IFN-α treatment reduced upstream pattern-recognition stimulation; however, this interpretation remains indirect because TLR7 activation, IRF7 phosphorylation, IFN secretion, and IFNAR/JAK-STAT signaling were not measured.

It is important to distinguish IFN induction from IFN effector signaling. Viral RNA recognition through TLR7/MyD88/IRF7 can contribute to type I IFN induction, whereas secreted IFN-α mediates antiviral effects primarily through IFNAR-dependent JAK-STAT signaling and downstream ISGs such as Mx. The observed transcriptional decrease in TLR7, MyD88, IRF7, and Mx after IFN-α treatment may therefore be consistent with reduced viral stimulation rather than direct negative regulation of the upstream pathway by IFN-α. Recent studies also show that host-directed antiviral outcomes are shaped by broader regulatory processes, including endolysosomal trafficking and metabolic or redox checkpoints [23,24]. Further protein-level and functional-dependence experiments are needed before the complete antiviral pathway can be defined.

## 5. Limitations and Future Work

This study has several limitations. First, the conclusions are based primarily on qRT-PCR analysis of transcript abundance; protein-level evidence for pathway activation, including IRF7 phosphorylation or nuclear translocation, secreted IFN-α, IFNAR abundance, downstream JAK-STAT activation, and loss-of-function validation, was not obtained. Second, recombinant proteins were produced in *E. coli*, and endotoxin quantification or removal was not documented in the current experiments. Third, antiviral activity was assessed mainly by NDV NP transcript abundance and qualitative CPE observation; infectious-virus titration, plaque assay, immunofluorescence, and viral-protein immunoblotting remain necessary to confirm effects on infectious virus production. Fourth, detailed mechanistic analyses focused on IFN-α, whereas comparable analyses of recombinant Mx were not performed. Finally, chicken embryos and DF-1 cells are surrogate systems, and species-specific differences in avian type I IFN activity cannot be excluded. Future work should address these points using protein-level assays, endotoxin-controlled recombinant proteins, direct infectious-virus measurements, and pathway-interference experiments.

## 6. Conclusions

Recombinant IFN-α and Mx proteins from *L. ridibundus* were prepared and evaluated in NDV-related experimental systems. NDV infection was associated with increased IFN-α and Mx transcription in primary gull lymphocytes, and recombinant IFN-α was associated with reduced NDV NP transcript abundance in chicken embryos, DF-1 cells, and gull lymphocytes under the tested conditions. These findings support further investigation of gull IFN-α-mediated antiviral responses against NDV, but protein-level pathway validation and direct infectious-virus assays are required to confirm the mechanism.

## Figures and Tables

**Table 1:** The primer sequences and fluorescent quantitative primers for the genes.

Gene	Primers	Products (bp)
IFN-α	F1: GAATTCATGCCTGCGCCCGCAACCCACCAC R1: CTCGAGCTAATTGCACATGGTGCGGGTGAG	579
Mx	F2: GAATTCATGAATAATAAGAGTGGAGCGT R2: CTCGAGCTAATAAAAACAGCTTAGTATGACA	1161
β-actin-cells	β-Actin F: ACT ACC TCA TGA AGA TCC TGA CA β-Actin R: TCT CCT TCT GCA TCC TGT C	394
NDV NP	NDV NP F: AAC AAA CCA CTC AGG CAA G NDV NP R: CTG TGC TCT CTC TTC AGA CAC	189
β-actin-chicken embryos	Actin-F: GCGTGACATCAAGGAGAA Actin-R: AGCACTGTGTTGGCATAC	270
TLR7	TLR7 F: ATT TCT GGG ATG TGT GGT AT TLR7 R: TGA ATG CTC TGG GAA AGG T	118
MyD88	MyD88 F: TCG CAA ATT GTA ATG AAC CG MyD88 R: CTG GAA AGT GAT GAG TGT GA	143
IFN-γ	IFN-γ F: CAG CTC CAA GAA AGT AAA AGA C	155
Mx-1	Mx F: TTA TCG CCT ACA AAG AGA GAC ACT CAA Mx R: CTG CCC ACG ACA CTT CAC A	236

**Table 2:** Real-time fluorescence quantitative PCR reaction system.

Reagent	Volume (μL)
2 × SuperReal PreMix Plus (SYBR Green)	10
F	0.8
R	0.8
cDNA template	2
ddH_2_O	6.4

**Table 3:** Real-time fluorescence quantitative PCR reaction program.

Temperature	Time	
95 °C	15 min	
95 °C	10 s	40 cycle
60 °C	30 s	

## Data Availability

The data supporting the findings of this study are included in the article. Further inquiries can be directed to the corresponding authors.
